# The Sleep Revolution – a model for other disease assessment and follow-up

**DOI:** 10.1192/j.eurpsy.2025.80

**Published:** 2025-08-26

**Authors:** E. S. Arnardóttir

**Affiliations:** 1 Reykjavik University, Reykjavik, Iceland; 2 European Sleep Research Society, Regensburg, Germany

## Abstract

**Abstract:**

In this talk, Dr. Arnardottir will summarize the main findings of the SLEEP REVOLUTION, an EU Horizon 2020 Research and Innovation Programme no. 965417. The project is an inter-disciplinary and international research and development project with 39 partner institutions and companies in Europe and Australia. SLEEP REVOLUTION aims to introduce an approach based on machine learning to assess sleep apnea severity and treatment needs as well as the use of digital health technology to measure sleep and health (app, cognitive testing, consumer sleep technology and clinically validated sleep technology for home use). Through these technological solutions , the project brings together researchers, patients and healthcare professionals to provide beyond the current state-of-the-art diagnosis and optimal treatment for sleep disorders. The approach of this project can be used as a model for other disease assessment and follow-up.

**Disclosure of Interest:**

E. Arnardóttir Consultant of: Medical Advisory Board for Philips (Feb 2022-Feb 2024) and Lille (Sept 2024), Speakers bureau of: Nox Medical, ResMed, Jazz Pharmaceuticals, Linde Healthcare, Apnimed, Wink Sleep, Vistor (NovoNordisk)

